# Absence of Glaucoma in Tg-*MYOC^Y437H^* Mice of Diverse Genetic Backgrounds

**DOI:** 10.1167/iovs.66.12.40

**Published:** 2025-09-18

**Authors:** Nicholas G. Tolman, Marina Simón, Felicia A. Juarez, Chi Zhang, Zhivka I. Hristova, Tionna B. Ouellette, Michael A. Sellarole, Wilhelmine N. deVries, Christa Montgomery, Simon W. M. John

**Affiliations:** 1 Department of Ophthalmology, Vagelos College of Physicians and Surgeons, Columbia University Irving Medical Center, New York, New York, United States; 2 The Jackson Laboratory, Bar Harbor, Maine, United States; 3 Zuckerman Mind Brain Behavior Institute, Columbia University, New York, New York, United States

**Keywords:** myocilin, MYOC, glaucoma, intraocular pressure (IOP)

## Abstract

**Purpose:**

Mutations in the myocilin (*MYOC*) gene cause elevated intraocular pressure and glaucoma. To better understand the factors influencing susceptibility to glaucoma, we studied the *MYOC* mutation (*MYOC^Y437H^*).

**Methods:**

We characterized ocular phenotypes in *Tg-MYOC^Y437H^* mice on nine different mouse strain backgrounds.

**Results:**

No glaucoma-related phenotypes were observed. We detected neither elevated intraocular pressure (daytime readings) nor optic nerve degeneration differences between wild type (WT) and *Tg-MYOC^Y437H^* mice. This included an absence of *Tg-MYOC^Y437H^*–induced phenotypes on a mixed B6SJL background that best reflected the original publications with this strain. We confirmed that this result was not due to an absence of transgene expression in ocular tissues.

**Conclusions:**

Our data indicate undefined complexity and that the previously reported glaucoma phenotypes are not robust across all institutions and environments.

Mutations in the myocilin gene (*MYOC*) are known to cause both juvenile glaucoma and adult-onset primary open-angle glaucoma (POAG).^1^^–^^4^ These mutations lead to elevated intraocular pressure (IOP), a key risk factor for glaucoma. The mutations compromise aqueous humor drainage from the eye by damaging trabecular meshwork (TM) cells, which are a key component of the ocular outflow pathway.^5^^–^^7^ Close to 100 different pathogenic *MYOC* mutations have been identified, and the associated disease phenotypes vary in severity and age of onset. In addition, there is variable penetrance and expressivity of disease associated with individual *MYOC* mutations,^1^ suggesting that genetic background and/or other factors have an important influence on outcomes. Pathogenic mutations primarily cluster in the protein's olfactomedin domain, but the significance of this mutation distribution is unclear.^2^ Various observations in humans and animal studies of myocilin indicate that various mutations act via a gain-of-function.^8^^–^^13^ Mutations elevate IOP by driving protein misfolding and impeding secretion of *MYOC* from the cell, resulting in intracellular accumulation of *MYOC* in TM cells.^14^ Although intracellular accumulation alone is not sufficient to elevate IOP in mice,^15^ it is suggested to lead to the progressive death of TM cells. *MYOC* mutations can also impact the morphogenesis of the ocular drainage tissues.^4^^,^^13^^,^^16^

A commonly studied pathogenic mutation in *MYOC* causes a *Y437H* substitution in the protein's olfactomedin domain. This *MYOC^Y437H^* mutation is associated with relatively severe disease outcomes, with patients commonly developing IOP elevation and glaucoma in the second decade of life.^17^ Mice that express the human *MYOC^Y437H^* mutant protein in TM cells have been reported to develop elevated IOP and glaucoma similar to patients with POAG.^8^^,^^18^ However, certain genetic backgrounds with the *MYOC^Y437H^* mutant protein have proven to be resistant to IOP elevation and glaucoma.^18^ In addition, mice with the equivalent mutation in their endogenous *Myoc* locus (*Myoc^Y423H^*) across four different strain backgrounds do not develop IOP elevation and glaucoma, despite impeded secretion of the mutant protein.^15^ Together, these results indicate that there are various unknown factors (e.g. genetics and environmental) that influence susceptibility to IOP elevation and glaucoma in *MYOC^Y437H^* mutant models.

To further interrogate mechanisms underlying IOP elevation in this POAG model, we imported mice with a transgene expressing the *MYOC^Y437H^* allele (*Tg-MYOC^Y437H^*).^8^ On the original mixed genetic background (C57BL/6J × SJL/J mix, henceforth called B6SJL, see the Methods section), we did not detect glaucoma-associated phenotypes in *Tg-MYOC^Y437H^* mice. As we and others have shown that strain background can have a major impact on IOP and glaucoma phenotypes,^18^^–^^20^ we decided to transfer the mutation to genetically diverse strain backgrounds. We hypothesized that altering strain background may reveal a robust glaucoma phenotype in *Tg-MYOC^Y437H^* mice. To test this, we analyzed the *Tg-MYOC^Y437H^* on nine different strain backgrounds, including the segregating B6SJL strain background on which this allele was originally published.^8^ Surprisingly, we did not detect any glaucoma-related phenotypes in *Tg-MYOC^Y437H^* mice on any examined background. These results show that the *Tg-MYOC^Y437H^* allele does not robustly produce glaucoma phenotypes in all laboratories.

## Methods

### Animal Husbandry and Ethics Statement

We imported B6SJL;Tg(CMV-*MYOC^Y437H^*)/Vcs mice as a generous gift from Dr. Val Sheffield's laboratory (University of Iowa). The Tg(CMV-MYOC*Y437H)#Vcs (*Tg-MYOC^Y437H^*) allele was generated by cloning the human MYOC gene into the mammalian expression vector pCS^+^ and a mutagenesis kit was used to introduce the Tyr437His mutation.^8^ The expression cassette containing a CMV promotor and *MYOC* cDNA was injected into the pronucleus of freshly fertilized C57BL/6J x SJL/J (B6SJL) oocytes.^8^ Founder males were mated to C57BL/6J mice before being intercrossed to generate B6SJL;Tg(CMV-MYOC^Y437H^)/Vcs mice.^8^

Although the original genetic background that we received was randomly mixed, we refer to it as B6SJL to reflect its genetic components from both the C57BL/6J and SJL/J strains. We harvested sperm from B6SJL mice and used it to in vitro fertilize C57BL/6J and DBA/2J oocytes. This generated two separate F1 cohorts (called B6xB6SJL F1 and D2xB6SJL F1 mice). We also directly backcrossed the *MYOC^Y437H^* allele from B6SJL mice to the A/J (AJ, Stock# 000646), C57BL/6J (B6J, Stock# 000664), C3A/BLiA-*Pde6b^+^*/J (C3A, Stock# 001912), and SJL/J strain backgrounds (SJL, Stock# 000686) for at least 6 and up to 11 generations. The *Tg-MYOC^Y437H^* allele was also backcrossed to strain DBA/2J (Stock# 00671) for 8 generations before crossing to the non-glaucomatous DBA/2J-*Gpnmb^+^/*SjJ substrain (D2-G, Stock# 007048) for 3 additional generations and generated a *Gpnmb^+/+^* experimental cohort. In addition, we backcrossed the *Tg-MYOC^Y437H^* allele to the C57BL/6NJ-*Crb1^rd8+em1Mvw^*/MvwJ (B6N, Stock# 022521) background, which contains a corrected *rd8* allele, for 10 generations. Additionally, we maintained the mutation on the original mixed genetic background by intercrossing and analyzing B6SJL mutant mice at intercross generations 1 to 7. No differences in phenotypes were found between any of these B6SJL intercross generations, so we combined all mice for analysis. As a positive glaucoma control, we included data from 12-month-old DBA/2J (Stock# 00671) mice collected over an overlapping timeframe as *Tg-MYOC^Y437H^* mice. DBA/2J mice develop robust IOP elevation and glaucoma.^21^^–^^23^ Given ongoing negative results, our move to a new institution, and the coronavirus disease (COVID) pandemic, some backgrounds were only analyzed for IOP or otherwise analyzed in less depth (e.g. B6N).

All of the mice in our experiments were maintained on NIH 31 (6% fat) diet ad libitum with HCl acidified water (pH 2.8–3.2). *Tg-MYOC^Y437H^* and wild type (WT) littermates were housed together. All mice were housed with pine wood shavings as bedding and covered with polyester filters, as previously described.^22^^,^^24^ Cages were maintained in an environment kept at 21°C with a 14-hour light: 10-hour dark cycle. Cohorts included a balanced number of male and female mice. All mice were treated in accordance with the Association for Research in Vision and Ophthalmology's statement on the use of animals in ophthalmic research. Live animal experiments were conducted at the Jackson Laboratory. The Institutional Animal Care and Use Committee of The Jackson Laboratory approved all experimental protocols. Data generation from harvested samples, data analyses, and manuscript preparation were performed at Columbia University.

### Genotyping of the Tg-MYOC^Y437H^ Allele

The genotype of the *Tg-MYOC^Y437H^* transgene was determined using a PCR protocol. Genomic DNA was PCR amplified with a forward 5′-CGTGCCTAATGGGAGGTCTAT-3′ and reverse 5′-CTGGTCCAAGGTCAATTGGT-3′ primer set specific for the *Tg-MYOC^Y437H^* allele. Genomic DNA was PCR amplified using the following program: (1) 93°C for 3 minutes, (2) 93°C for 15 seconds, (3) 63°C for 1 minute, (4) 72°C for 45 seconds, (5) repeat steps 2 to 4 for 35 times each, and (6) 72°C for 5 minutes. Then, 5 µL of sample was run on a 3% agarose gel. The *Tg-MYOC^Y437H^* allele amplifies a 453-base pair fragment.

### IOP Measurement and Anterior Chamber Depth

IOP was measured during the light part of the day using the microneedle method, as previously described.^25^^,^^26^ Mice were acclimatized to the procedure room and anesthetized via an intraperitoneal injection of a mixture of ketamine (99 mg/kg; Ketlar, Parke-Davis, Paramus, NJ, USA) and xylazine (9 mg/kg; Rompun, Phoenix Pharmaceutical, St. Joseph, MO, USA) immediately prior to IOP assessment, a procedure that does not alter IOP in the experimental window.^26^ During each IOP measurement period, eyes of independent WT B6 mice were assessed in parallel with experimental mice as a methodological control to ensure proper calibration and equipment function. For the B6/B6SJL and D2/B6SJL F1 cohorts, IOP was measured at 6, 9, and 12 months of age, with all mice within a 2-week age window around those ages. For all other background strains, IOPs were measured at a young timepoint (3–4 months of age), an intermediate timepoint (6–8 months of age), and an older timepoint (12–14 months of age). All cohorts included a balanced number of male and female mice. We compared IOP distributions within all strain backgrounds by 2-way ANOVA, with age and *Tg-MYOC^Y437H^* genotype as independent variables. IOPs of B6N background mice were only compared at one age (12–15 months) and across *Tg-MYOC^Y437H^* genotype. Differences between genotype groups were compared by Tukey's honestly significant difference test. We measured IOP of typically 30 to 50 eyes and at least 25 eyes per group (age, genotype, and strain background) except for SJL background mice at 12 to 14 months where 12 transgenic and 13 WT eyes were examined, and B6N background mice where 53 transgenic and 12 WT eyes were examined.

### Anterior Chamber Examination

As anterior chamber depth is a sensitive indicator of exposure to high IOP in mice,^20^^,^^27^^,^^28^ even if the pressure is high at a different time or day or was elevated at an earlier age, we also evaluated the depth of anterior chamber in all eyes. Anterior eye tissues were examined approximately every 3 months between 3 and 15 months of age. Using a slit-lamp biomicroscope, we evaluated all mice for phenotypic abnormalities associated with IOP elevation, including pupillary abnormalities, generalized corneal haze, buphthalmos, and deepening of the anterior chamber, in 10 to 20 eyes from *Tg-MYOC^Y437H^* mice on AJ, D2-G B6J, and C3A backgrounds at 12 to 14 months of age. Only female mice were used for slit-lamp examination. In addition, mice of all nine genetic backgrounds had their anterior chambers evaluated under a dissection microscope using drop PBS on the cornea to provide a clear view into the eye while performing IOP measurements (same ages as above). No differences in phenotypic abnormalities were observed between the methods.

### Optic Nerve Assessment

Intracranial portions of optic nerves were dissected, processed, and analyzed as previously described.^29^^–^^32^ Briefly, optic nerve cross-sections were stained with para-phenylenediamine (PPD) and examined for glaucomatous damage. PPD stains all myelin sheaths of a healthy axon, but differentially darkly stains the myelin sheaths and the axoplasm of sick or dying axons. This allows for the sensitive detection and quantification of axon damage and loss. Significant optic nerve axon loss was previously reported at 4 to 5 months in *Tg-MYOC^Y437H^* mice, with substantial loss by 12 to 14 months.^8^ As we detected no abnormal anterior chamber depth or IOP phenotypes in our mice, we analyzed optic nerves primarily at ages ≥ 12 months (with the majority at ∼ 12 months with a limited number at older ages out to 17 months) for both WT and *Tg-MYOC^Y437H^* mice of all examined backgrounds. As glaucomatous neurodegeneration was not detected at any age, we combined data for all ages.

Degree of damage was assigned using a damage rating scale that is well-validated against axon counting.^30^^,^^33^ Multiple sections of each nerve were considered when determining the damage level. One of three damage levels was assigned:

(1) No damage/glaucoma (NO) – less than 5% axons damaged and no gliosis. This level of damage is seen in age- and sex-matched non-glaucomatous mice and is not due to glaucoma.
(2) Moderate damage (MOD) – average of 30% axon loss and early gliosis.
(3) Severe (SEV) – > 50% axonal loss and damage with prominent gliosis.

For each background and group, we analyzed 18 to 34 nerves except for C3A and D2 WT mice (*n* = 17 and 12, respectively) and SJL mice. As SJL mice naturally develop retinal degeneration as a consequence of the *Pde6b^rd1^* mutation,^34^ and the effects on glaucoma are uncertain, we restricted examination to a small cohort of 6 Tg and 8 WT nerves. All examined F1 B6SJL mice were either heterozygous or WT for the *Pde6b^rd1^* allele and so did not develop retinal degeneration. We discarded a small number of both WT and Tg nerves of the B6SJL F1 background from analysis because of very severe segmental damage with swelling resembling ischemia, possibly due to potential stroke and inflammatory risk in this background or some unknown trauma that may have occurred.^35^^,^^36^ Because of a laboratory move and the lack of detected anterior chamber deepening and IOP elevation, we discontinued examining B6N background mice without examining the nerves. Age- and sex-matched WT nerves of all other genetic backgrounds were analyzed as above.

### Optic Nerve Axon Counting

We selected four backgrounds (AJ, B6J, B6SJL, and C3A) for axon counting using the AxonJ software in ImageJ, as previously reported.^37^ This software enables reproducible axon counting from PPD-stained optic nerve images by applying edge detection, morphological filters, and Hessian-based convolution to distinguish axons from the background. The method has been extensively validated against expert manual counts, demonstrating high accuracy and reproducibility. We randomly selected 3 high-quality sections (technical replicates) per nerve and counted axons within one large rectangular region per section with each rectangle sampling ≈ 50% of the nerve area. These sections were imaged using a Keyence BZ-X810 microscope using the brightfield settings and a 100 × 1.45 NA oil objective. We used AxonJ to count axons twice per section to mitigate seed point variation. To calculate total axons per section, we extrapolated the axon count within each rectangular region to the total area of the nerve in each section and averaged the two measurements for each section. The total axon count for all three sections was then averaged to produce the axon number per nerve. We counted axons for at least 9 and up to 19 nerves for any experimental genotype group, with matched numbers of mice of each genotype belonging for any strain background. Nerves were randomly selected from each group. A small number of randomly selected nerves were not appropriately stained to be reliable for automated counting and were replaced. The number of axons was compared by ANOVA followed by Tukey's honestly significant difference test.

### MicronIV and Image-Guided Optical Coherence Tomography

Optical coherence tomography (OCT) paired with the MicronIV: Retinal Imaging Microscope (Phoenix Research Labs) was used to assess the retina and optic nerve head in vivo. Mice were subject to pupillary dilation with a drop of 1% solution of cyclopentolate hydrochloride ophthalmic solution (Akorn, Inc.) that was topically applied to the cornea. Mice were anesthetized as previously described (see the Methods section, IOP measurement). Mice were secured on a rotating stage and imaged using OCT per the manufacturer's directions. From all tested backgrounds, we examined at least 8 eyes per genotype at 10 to 12 months of age. B6xB6SJL F1 and D2xB6SJL F1 cohorts were analyzed prior to OCT equipment being available. Due to a laboratory move and lack of IOP elevation, the B6N background cohort was not examined.

### RNA Isolation, cDNA Synthesis, and RT-PCR

We examined expression of the mouse *Myoc* and human *Tg-MYOC^Y437H^* alleles in eyes of each genotype and strain background between 3 and 7 months of age. For whole eye tissue, fresh postmortem enucleated eyes minus the lens were analyzed. The lens was removed after making a small incision in the cornea. All remaining tissue was immediately placed in RNA later solution (Ambion). RNA was isolated and processed for RT-PCR using the PureLink RNA Mini Kit (ThermoFisher). Total RNA was reverse transcribed using the Verso 1-Step RT-PCR Kit ReddyMix, with ThermoPrime Taq (ThermoFisher). A portion of the cDNA was then used in three separate PCR reactions to detect endogenous mouse *Myoc* (*mMYOC*), *Tg-MYOC^Y437H^* (*hMYOC*), and *Gapdh* as a control. For *mMyoc*, we used a primer set: forward 5′-GCCATCCAAGACCTTCAGAG-3′ and reverse 5′-AGATCCCTGGTTTGGGTCTC-3′. For *Gapdh*, we used a primer set: 5′-ACCACAGTCCATGCCATCAC-3′ and reverse 5′-TCCACCACCCTGTTGCTGTA-3′. For *hMYOC*, we used a primer set: forward 5′-GAGTAAGGCAAGAAAATGAGAATC-3′ and reverse 5′-CCTCTCCACTCCTGAGATAGC -3′. For *mMyoc* and *Gapdh*, cDNA was amplified using the following program; (1) 94°C for 3 minutes, (2) 94°C for 30 seconds, (3) 60°C for 30 seconds, (4) 72°C for 1 minute, (5) repeat steps 2 to 4 for 39 times, and (6) 72°C for 5 minutes. The *hMYOC* cDNA was amplified using the following program: (1) 94°C for 3 minutes, (2) 94°C for 30 seconds, (3) 53°C for 1 minute, (4) 72°C for 1 minute + 1 second/cycle extension, (5) repeat steps 2 to 4 for 39 times, and (6) 72°C for 5 minutes. Then, 5 µL of sample was run on a 3% agarose gel. The *mMyoc* allele amplifies a 206 base pair fragment, the *hMYOC* allele amplifies a 249 base pair fragment, and the *Gapdh* allele amplifies a 452 base pair fragment. We analyzed a total of 12 mice of each genotype across all genetic backgrounds (24 eyes). Despite random variability in the amount of amplified product, the results clearly identified expression of the mutant transgene in all *Tg-MYOC^Y437H^* eyes.

## Results

### No Detectable IOP Elevation or Glaucoma in Tg-MYOC^Y437H^ Mice

**Figure 1.**
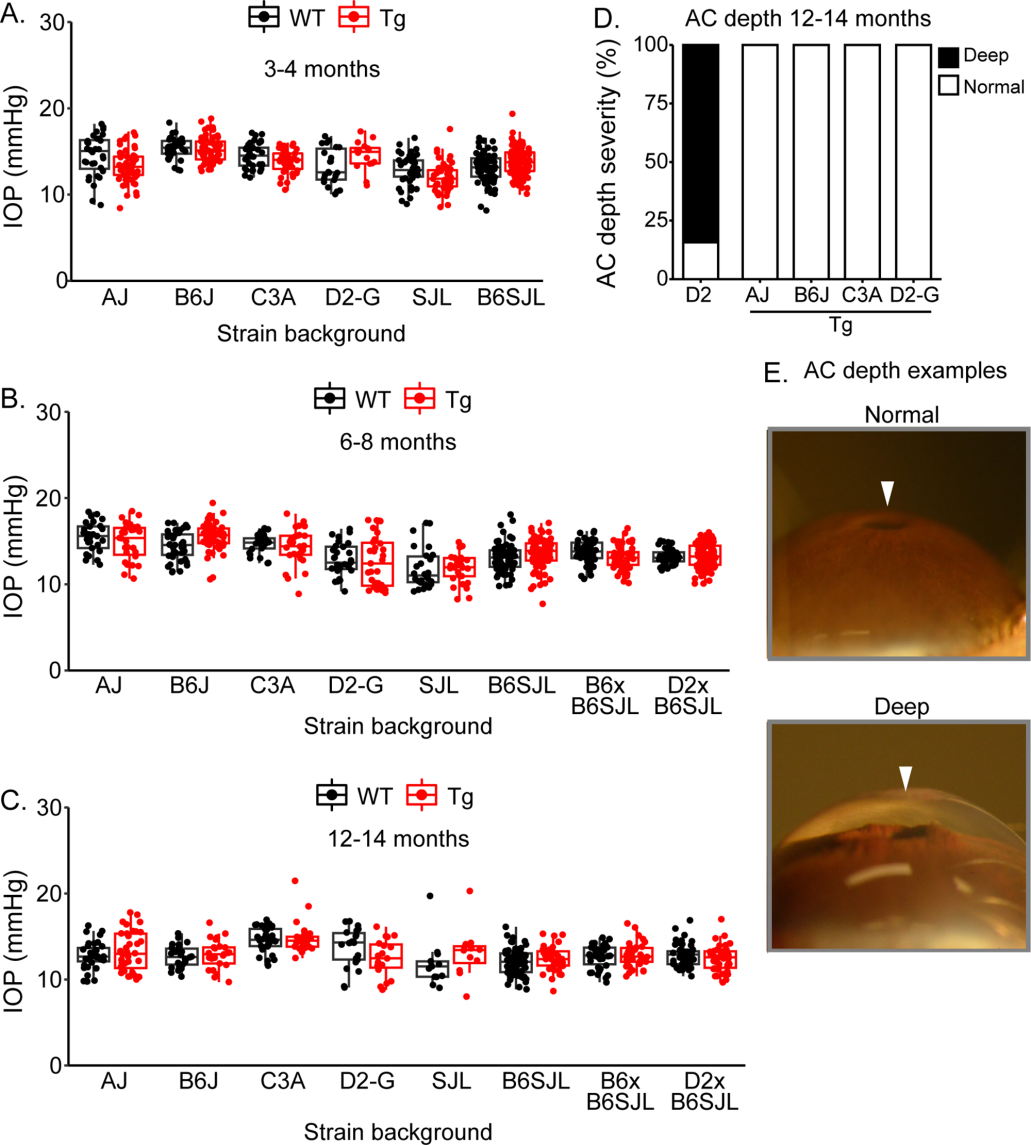
**IOP in *Tg-MYOC^Y437H^* mice with different genetic backgrounds.** (**A–C**) Boxplots of IOP (interquartile range and median line). *Tg-MYOC^Y437H^* genotype did not significantly change IOP (age- and strain-matched, all *P* > 0.06, even SJL at 12–14 months which was lowly sampled, *P* > 0.5). (**D**) In agreement, no anterior chamber deepening was found in *Tg-MYOC^Y437H^* mice across backgrounds. Age-matched DBA/2J (D2, established glaucoma model) data are shown as a positive control for pathogenic AC deepening. There were 12 to 20 eyes examined in each *Tg-MYOC^Y437H^* group. (**E**) Examples of AC depth severities with the deep AC image of a positive control eye.

**Figure 2.**
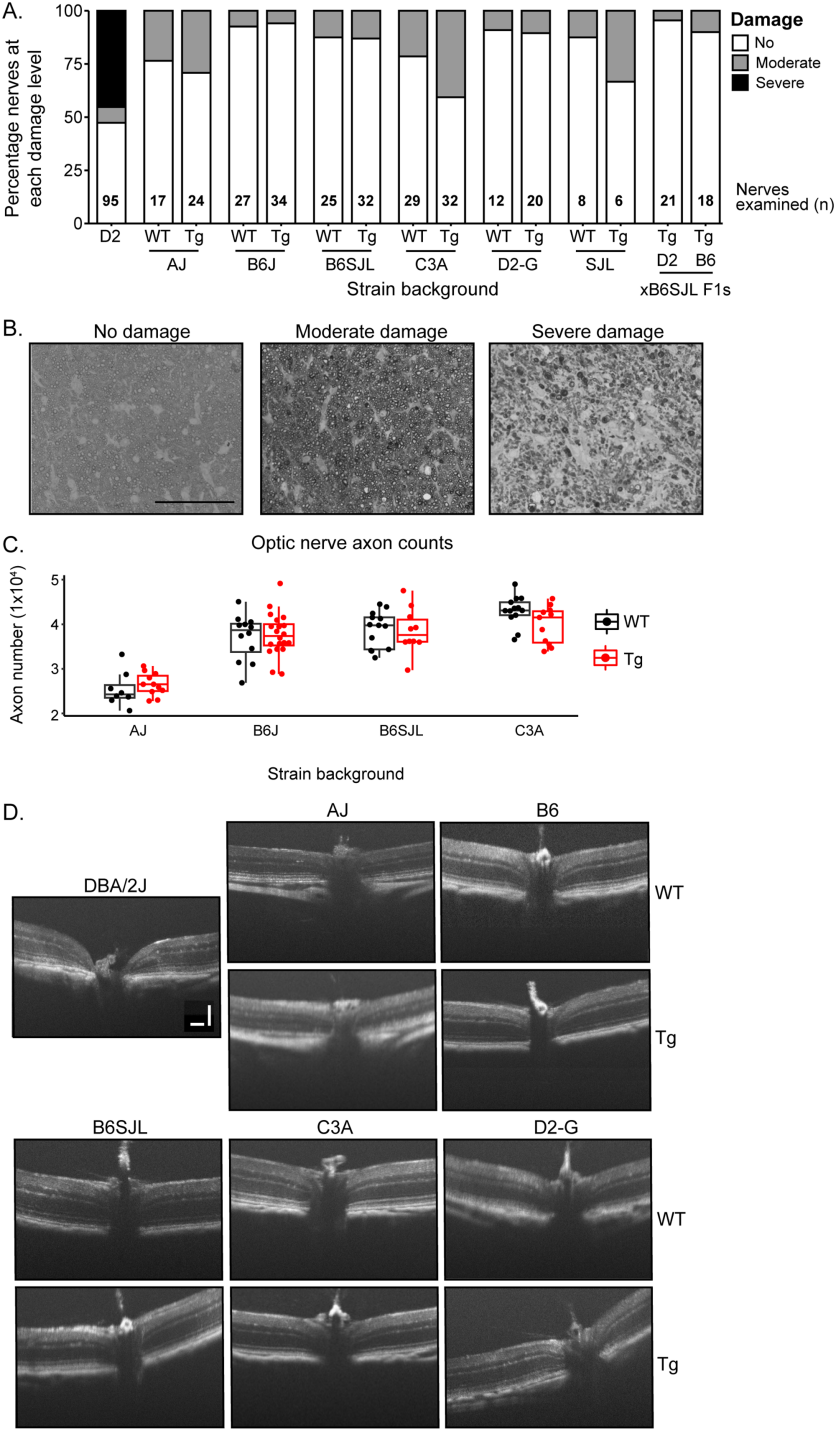
**No significant optic nerve health changes in *Tg-MYOC^Y437H^* mice.** (**A**) Frequency histogram of optic nerve damage evident in PPD-stained cross sections of 10 to 17-month-old nerves (majority 12–14 months, number of nerves examined shown in figure). Twelve-month-old DBA/2J (D2) mice, an established glaucoma model, show a high incidence of severe glaucoma. Moderate damage, likely due to stresses with age, occurs in some nerves of WT and *Tg-MYOC^Y437H^* eyes (Fisher's exact test, all *P* > 0.3) that were not detected to have high IOP or deep anterior chambers, whereas the subset analyzed had no optic nerve excavation by OCT. Although not significantly convincing between *Myoc* genotypes, there was a trend to a greater occurrence of this damage in the reasonably sampled A/J and C3A strain backgrounds, but this difference could be due to chance sampling. Deeper sampling would be required to determine if this is due to genotype or chance. (**B**) Representative images of PPD-stained optic nerve cross sections for different severity levels. *Scale bar* = 100 µm. (**C**) Boxplots (interquartile range and median line) of the total number of axons in *Tg-MYOC^Y437H^* and WT optic nerve cross sections. There were no significant changes in axon numbers between genotypes (all *P* > 0.6). The majority of nerves counted were 12 to 14 months and were age-matched across genotypes. AJ nerves were typically analyzed at an older age compared with other groups (14 months). (**D**) Representative SD-OCT images of the retina and optic nerve head at 10 months of age. WT and *Tg-MYOC^Y437H^* eyes of each strain background have a normal nerve head. *Scale bar* = 100 µm. SD-OCT, spectral-domain optical coherence tomography.

We first longitudinally characterized IOP in *Tg-MYOC^Y437H^* and WT control mice (lacking the *MYOC* transgene) of all nine strain backgrounds. We detected no IOP changes between *Tg-MYOC^Y437H^* mice compared with WT mice (Figs. 1A–C, Supplementary Fig. S1). In addition to IOP measurements, we inspected eyes by slit-lamp, confirming the lack of abnormal anterior segment phenotypes in *Tg-MYOC^Y437H^* eyes (Figs. 1D, 1E). Importantly and confirming the IOP findings, the anterior chamber depth was normal in all mice at all ages at which IOP was assessed. This is important because increases in anterior chamber depth are a sensitive and indelible indicator of even modestly elevated IOP in mice.^20^^,^^27^^,^^28^ This allows detection of eyes that have been exposed to high IOP even if the IOP elevation occurred at a different time of day or night to the IOP measurement or occurred at a different age or timepoint. Finally, we examined the optic nerves and retinas of WT and *Tg-MYOC^Y437H^* mice (Figs. 2A–D). For all strain backgrounds, *Tg-MYOC^Y437H^* caused no glaucoma, with no significantly different degree or occurrence of damage beyond non-*MYOC^Y437H^* related differences (age- and or other strain-dependent damage) that were present in WT mice (see the Methods section). This lack of damage is seen in both OCT imaging and in histological examination of optic nerve cross sections. Finally, we counted axon numbers, detecting no significant differences between WT and *Tg-MYOC^Y437H^* nerves.

### Loss of Tg-MYOC^Y437H^ Expression Is not Responsible for the Lack of Glaucoma Phenotypes

Because no IOP or glaucoma phenotypes were detected despite our analyzing a large number of mice with diverse genetic makeups, we decided to confirm that *MYOC^Y437H^* transgene was still being expressed in ocular tissues. On each strain background, expression of the *Tg-MYOC^Y437H^* transcript was readily detected in mutant but not in WT mice (Supplementary Fig. S2).

## Discussion

In our colony and environment, the *Tg-MYOC^Y437H^* allele did not cause either high IOP or glaucoma on any of the nine genetic backgrounds (including F1s) that were aged and analyzed. This is despite our confirming that the mutant gene is expressed and has not undergone some form of spontaneous inactivation. It remains possible that the transgenic allele has changed in some way to explain the differences. However, this seems unlikely as to account for the lack of glaucoma on all strain backgrounds it would either: (1) have had to happen very early in the alleles history, as we obtained the mice prior to the initial publication that introduced it; or (2) it would have had to independently change on different tested genetic backgrounds as independent founder mice were used to establish colonies on different genetic backgrounds. Although independent changes on each of multiple backgrounds seem unlikely, it may be that there was a single common change if the founders shared a common ancestor in which the allele had changed soon after its generation at Iowa and prior to shipping to us, and that these “changed” mice were then not used to propagate the strain at Iowa, but we cannot assess that. Further arguing against the above changes, an independent group recently reported no IOP or aqueous humor drainage abnormalities in *Tg-MYOC^Y437H^* mice separated from the original colony at a later date.^38^

Although we did not detect glaucoma or increased axon loss compared with strain matched controls in *Tg-MYOC^Y437H^* nerves, we did find age-related damage on all backgrounds, distributed across WT and *Tg-MYOC^Y437H^* genotypes. C3A, AJ, and SJL *Tg-MYOC^Y437H^* eyes had a higher proportion of age-related damage compared with WT nerves (but SJL had very low sample sizes, all *P* > 0.3). Although not statistically significant, it is possible that the transgene is influencing age-related or other pathologic changes not related to high IOP on these backgrounds, but much deeper sampling would be needed to determine this. In addition, it remains possible that glaucoma-associated phenotypes could be detected on additional untested strain backgrounds or with further aging in our environment. Another laboratory has independently failed to detect the glaucoma-inducing high IOP in *Tg-MYOC^Y437H^* mice.^38^ The discrepancy in phenotypes between laboratories strongly argues that the transgene does not produce glaucoma in all settings. The reasons for this are not clear and must involve environmental factors, as we have included genetic contexts as close as possible to those previously reported to develop glaucoma. The nature of the important environmental factors remains unknown, but the presence or absence of specific gut bacteria alters other conditions (e.g. rheumatoid arthritis, asthma, and type 2 diabetes).^39^^–^^41^ Aligned with possible microbial differences, our mice were housed with acidified water that modulates microbial growth in their water bottles and could alter the microbiome. However, acidified water was not used in an independent study that also reported no changes in IOP, aqueous humor outflow facility (a direct and sensitive measure of ocular drainage tissue function), or TM cell density (used on 6–7-month-old *Tg-MYOC^Y437H^* mice of a B6SJL F1 background).^38^ If the microbiome is important, differing rederivation and housing conditions between institutes could alter the phenotypes. We are aware that other groups have not detected glaucoma but have not published their findings due to the inertia against publishing negative results, so it is not clear how commonly these mice fail to develop high IOP and glaucoma at different institutions. The type of diet and how it is sourced and treated, the type, treatment, and changing frequency of mouse bedding, the rate of cage air exchange/type of cage, and many other variables could have an impact. Substantial work would be required to resolve this.

## Supplementary Material

Supplement 1

## References

[1] Gong G, Kosoko-Lasaki O, Haynatzki GR, Wilson MR. Genetic dissection of myocilin glaucoma. *Hum Mol Genet*. 2004; 13 Spec No 1: R91–R102. 14764620 10.1093/hmg/ddh074

[2] Fingert JH, Stone EM, Sheffield VC, Alward WL. Myocilin glaucoma. *Surv Ophthalmol*. 2002; 47(6): 547–561. 12504739 10.1016/s0039-6257(02)00353-3

[3] Saccuzzo EG, Youngblood HA, Lieberman RL. Myocilin misfolding and glaucoma: a 20-year update. *Prog Retin Eye Res**.* 2023; 95: 101188. 37217093 10.1016/j.preteyeres.2023.101188 PMC10330797

[4] Sharma R, Grover A. Myocilin-associated glaucoma: a historical perspective and recent research progress. *Mol Vis**.* 2021; 27: 480–493. 34497454 PMC8403517

[5] Acott TS, Kelley MJ. Extracellular matrix in the trabecular meshwork. *Exp Eye Res**.* 2008; 86(4): 543–561. 18313051 10.1016/j.exer.2008.01.013 PMC2376254

[6] Buffault J, Labbe A, Hamard P, Brignole-Baudouin F, Baudouin C. The trabecular meshwork: structure, function and clinical implications. A review of the literature. *J Fr Ophtalmol*. 2020; 43(7): e217–e230. 32561029 10.1016/j.jfo.2020.05.002

[7] Overby DR, Stamer WD, Johnson M. The changing paradigm of outflow resistance generation: towards synergistic models of the JCT and inner wall endothelium. *Exp Eye Res**.* 2009; 88(4): 656–670. 19103197 10.1016/j.exer.2008.11.033 PMC2744486

[8] Zode GS, Kuehn MH, Nishimura DY, et al. Reduction of ER stress via a chemical chaperone prevents disease phenotypes in a mouse model of primary open angle glaucoma. *J Clin Invest**.* 2011; 121(9): 3542–3553. 21821918 10.1172/JCI58183 PMC3163970

[9] Kim BS, Savinova OV, Reedy MV, et al. Targeted disruption of the myocilin gene (Myoc) suggests that human glaucoma-causing mutations are gain of function. *Mol Cell Biol**.* 2001; 21(22): 7707–7713. 11604506 10.1128/MCB.21.22.7707-7713.2001 PMC99941

[10] Pang CP, Leung YF, Fan B, et al. TIGR/MYOC gene sequence alterations in individuals with and without primary open-angle glaucoma. *Invest Ophthalmol Vis Sci**.* 2002; 43(10): 3231–3235. 12356829

[11] Morissette J, Clepet C, Moisan S, et al. Homozygotes carrying an autosomal dominant TIGR mutation do not manifest glaucoma. *Nat Genet**.* 1998; 19(4): 319–321. 9697688 10.1038/1203

[12] Wiggs JL, Vollrath D. Molecular and clinical evaluation of a patient hemizygous for TIGR/MYOC. *Arch Ophthalmol*. 2001; 119(11): 1674–1678. 11709019 10.1001/archopht.119.11.1674

[13] Senatorov V, Malyukova I, Fariss R, et al. Expression of mutated mouse myocilin induces open-angle glaucoma in transgenic mice. *J Neurosci*. 2006; 26(46): 11903–11914. 17108164 10.1523/JNEUROSCI.3020-06.2006 PMC6674879

[14] Lynch JM, Li B, Katoli P, et al. Binding of a glaucoma-associated myocilin variant to the alphaB-crystallin chaperone impedes protein clearance in trabecular meshwork cells. *J Biol Chem**.* 2018; 293(52): 20137–20156. 30389787 10.1074/jbc.RA118.004325 PMC6311499

[15] Gould DB, Reedy M, Wilson LA, Smith RS, Johnson RL, John SW. Mutant myocilin nonsecretion in vivo is not sufficient to cause glaucoma. *Mol Cell Biol**.* 2006; 26(22): 8427–8436. 16954374 10.1128/MCB.01127-06 PMC1636791

[16] Hamanaka T, Kimura M, Sakurai T, et al. A histologic categorization of aqueous outflow routes in familial open-angle glaucoma and associations with mutations in the MYOC gene in Japanese patients. *Invest Ophthalmol Vis Sci**.* 2017; 58(5): 2818–2831. 28564705 10.1167/iovs.16-20646

[17] Kwon YH, Fingert JH, Kuehn MH, Alward WL. Primary open-angle glaucoma. *N Engl J Med**.* 2009; 360(11): 1113–1124. 19279343 10.1056/NEJMra0804630 PMC3700399

[18] McDowell CM, Luan T, Zhang Z, et al. Mutant human myocilin induces strain specific differences in ocular hypertension and optic nerve damage in mice. *Exp Eye Res**.* 2012; 100: 65–72. 22575566 10.1016/j.exer.2012.04.016 PMC3612883

[19] Linder CC . Genetic variables that influence phenotype. *ILAR J**.* 2006; 47(2): 132–140. 16547370 10.1093/ilar.47.2.132

[20] Tolman NG, Balasubramanian R, Macalinao DG, et al. Genetic background modifies vulnerability to glaucoma-related phenotypes in Lmx1b mutant mice. *Dis Model Mech**.* 2021; 14(2): dmm046953. 33462143 10.1242/dmm.046953 PMC7903917

[21] Libby RT, Anderson MG, Pang IH, et al. Inherited glaucoma in DBA/2J mice: pertinent disease features for studying the neurodegeneration. *Vis Neurosci*. 2005; 22(5): 637–648. 16332275 10.1017/S0952523805225130

[22] John SW, Smith RS, Savinova OV, et al. Essential iris atrophy, pigment dispersion, and glaucoma in DBA/2J mice. *Invest Ophthalmol Vis Sci**.* 1998; 39(6): 951–962. 9579474

[23] Anderson MG, Smith RS, Hawes NL, et al. Mutations in genes encoding melanosomal proteins cause pigmentary glaucoma in DBA/2J mice. *Nat Genet**.* 2002; 30(1): 81–85. 11743578 10.1038/ng794

[24] Chang B, Smith RS, Hawes NL, et al. Interacting loci cause severe iris atrophy and glaucoma in DBA/2J mice. *Nat Genet**.* 1999; 21(4): 405–409. 10192392 10.1038/7741

[25] John SW, Hagaman JR, MacTaggart TE, Peng L, Smithes O. Intraocular pressure in inbred mouse strains. *Invest Ophthalmol Vis Sci**.* 1997; 38(1): 249–253. 9008647

[26] Savinova OV, Sugiyama F, Martin JE, et al. Intraocular pressure in genetically distinct mice: an update and strain survey. *BMC Genet**.* 2001; 2: 12. 11532192 10.1186/1471-2156-2-12 PMC48141

[27] Fiedorowicz M, Welniak-Kaminska M, Swiatkiewicz M, et al. Changes of ocular dimensions as a marker of disease progression in a murine model of pigmentary glaucoma. *Front Pharmacol*. 2020; 11: 573238. 33013417 10.3389/fphar.2020.573238 PMC7500411

[28] Yang XL, van der Merwe Y, Sims J, et al. Age-related changes in eye, brain and visuomotor behavior in the DBA/2J mouse model of chronic glaucoma. *Sci Rep**.* 2018; 8(1): 4643. 29545576 10.1038/s41598-018-22850-4 PMC5854610

[29] Smith RS, John SWM, Nishina PM, Sundberg JP. *Systematic Evaluation of the Mouse Eye: Anatomy, Pathology, and Biomethods*. Boca Raton, FL: CRC Press; 2002.

[30] Howell GR, Libby RT, Jakobs TC, et al. Axons of retinal ganglion cells are insulted in the optic nerve early in DBA/2J glaucoma. *J Cell Biol**.* 2007; 179(7): 1523–1537. 18158332 10.1083/jcb.200706181 PMC2373494

[31] Williams PA, Harder JM, Foxworth NE, et al. Vitamin B(3) modulates mitochondrial vulnerability and prevents glaucoma in aged mice. *Science**.* 2017; 355(6326): 756–760. 28209901 10.1126/science.aal0092 PMC5408298

[32] Nair KS, Cosma M, Raghupathy N, et al. YBR/EiJ mice: a new model of glaucoma caused by genes on chromosomes 4 and 17. *Dis Model Mech**.* 2016; 9(8): 863–871. 27483353 10.1242/dmm.024307 PMC5007977

[33] Howell GR, Soto I, Zhu X, et al. Radiation treatment inhibits monocyte entry into the optic nerve head and prevents neuronal damage in a mouse model of glaucoma. *J Clin Invest**.* 2012; 122(4): 1246–1261. 22426214 10.1172/JCI61135 PMC3314470

[34] Chang B, Hawes NL, Hurd RE, Davisson MT, Nusinowitz S, Heckenlively JR. Retinal degeneration mutants in the mouse. *Vision Res**.* 2002; 42(4): 517–525. 11853768 10.1016/s0042-6989(01)00146-8

[35] Yang G, Kitagawa K, Matsushita K, et al. C57BL/6 strain is most susceptible to cerebral ischemia following bilateral common carotid occlusion among seven mouse strains: selective neuronal death in the murine transient forebrain ischemia. *Brain Res**.* 1997; 752(1–2): 209–218. 9106459 10.1016/s0006-8993(96)01453-9

[36] Pollinger B, Krishnamoorthy G, Berer K, et al. Spontaneous relapsing-remitting EAE in the SJL/J mouse: MOG-reactive transgenic T cells recruit endogenous MOG-specific B cells. *J Exp Med**.* 2009; 206(6): 1303–1316. 19487416 10.1084/jem.20090299 PMC2715069

[37] Zarei K, Scheetz TE, Christopher M, et al. Automated axon counting in rodent optic nerve sections with AxonJ. *Sci Rep*. 2016; 6: 26559. 27226405 10.1038/srep26559 PMC4881014

[38] Bahrani Fard MR, Chan J, Read AT, et al. Magnetically steered cell therapy for reduction of intraocular pressure as a treatment strategy for open-angle glaucoma. *Elife*. 2025; 13: RP103256. 40622256 10.7554/eLife.103256 PMC12234007

[39] Scher JU, Sczesnak A, Longman RS, et al. Expansion of intestinal Prevotella copri correlates with enhanced susceptibility to arthritis. *Elife*. 2013; 2: e01202. 24192039 10.7554/eLife.01202 PMC3816614

[40] Barcik W, Boutin RCT, Sokolowska M, Finlay BB. The role of lung and gut microbiota in the pathology of asthma. *Immunity**.* 2020; 52(2): 241–255. 32075727 10.1016/j.immuni.2020.01.007 PMC7128389

[41] Larsen N, Vogensen FK, van den Berg FW, et al. Gut microbiota in human adults with type 2 diabetes differs from non-diabetic adults. *PLoS One**.* 2010; 5(2): e9085. 20140211 10.1371/journal.pone.0009085 PMC2816710

