# Supplementary material for: Absence of Glaucoma in Tg-MYOCY437H Mice of Diverse Genetic Backgrounds
Source: Invest Ophthalmol Vis Sci. 2025 Sep 18;66(12):40. doi: 10.1167/iovs.66.12.40 (PMC12449821; doi:10.1167/iovs.66.12.40)
Supplement: Supplement 1 [file iovs-66-12-40_s001.pdf]

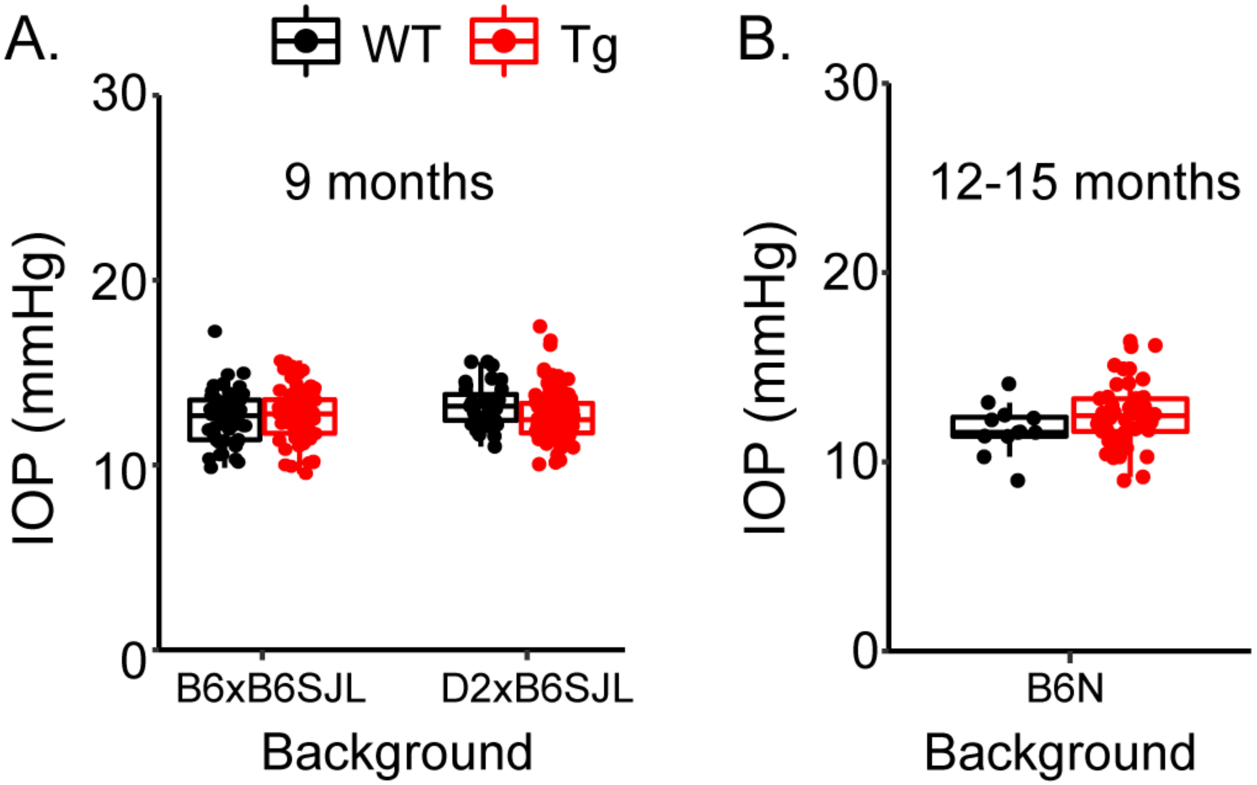


**Figure S1: No IOP elevation on other tested backgrounds. (A)** IOP (boxplots) does not change between WT and *Tg-MYOC*$^{Y437H}$ mice of B6xB6SJL or D2xB6SJL background at 9 months. (n > 30 eyes examined, P > 0.2). **(B)** No significant IOP change in B6N *Tg-MYOC*$^{Y437H}$ mice at 12-15 months age (n = 12 WT and n = 53 Tg eyes examined, *P* = 0.16).

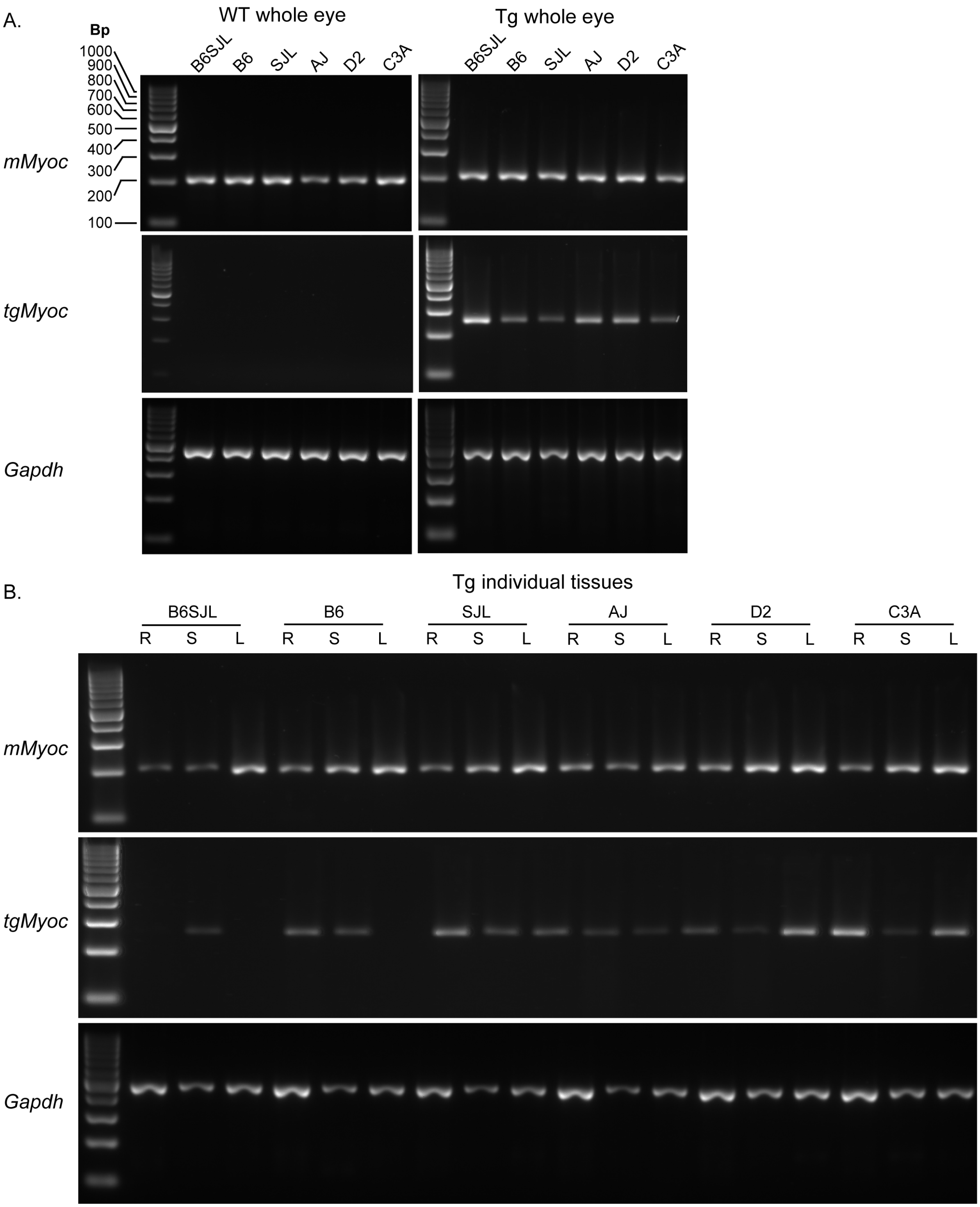


**Figure S2: RT-PCR gel images.** RT-PCR of whole eye tissue from 3-7 months old WT and *Tg-MYOC*$^{Y437H}$ mice The *Tg-MYOC*$^{Y437H}$ transcript is expressed in transgenic mice across strain backgrounds. Individual transcript's amplicon base pair (bp) size is compared against a DNA ladder.
